# Efficacy and Safety of Chaihu Jia Longgu Muli Decoction in the Treatment of Poststroke Depression: A Systematic Review and Meta-Analysis

**DOI:** 10.1155/2021/7604537

**Published:** 2021-08-19

**Authors:** Renhong Wan, Ruiwen Song, Yihua Fan, Linhui Li, Jiangxin Zhang, Beijia Zhang, Xinju Li, Shenjun Wang

**Affiliations:** ^1^Tianjin University of Traditional Chinese Medicine, Tianjin 301617, China; ^2^First Teaching Hospital of Tianjin University of Traditional Chinese Medicine, Tianjin 300193, China; ^3^National Clinical Research Center for Chinese Medicine Acupuncture and Moxibustion, Tianjin 300381, China; ^4^Research Center of Experimental Acupuncture Science, Tianjin University of Traditional Chinese Medicine, Tianjin, China; ^5^School of Acupuncture & Moxibustion and Tuina, Tianjin University of Traditional Chinese Medicine, Tianjin, China

## Abstract

**Objective:**

Chaihu Jia Longgu Muli decoction (CLMD) is widely used in the treatment of poststroke depression (PSD) in China. Some evidences show that it has advantages, but there lacks reliable evidence. This study aims to systematically evaluate the efficacy and safety of CLMD in the treatment of PSD.

**Methods:**

All randomized controlled trials (RCTs) of CLMD in the treatment of PSD were searched from the following databases: PubMed, Cochrane Library, Embase, Web of Science, China National Knowledge Infrastructure (CNKI), Wanfang Database, VIP Database, and Chinese Biomedical Literature Service System (CBM), from their inception to May 2021. Two researchers independently screened the literature, extracted the data, and evaluated the risk of bias in the included studies. Meta-analysis was performed using RevMan5.3 software.

**Results:**

A total of 13 RCTs involving 1665 patients were finally included in this study, among which 5 RCTs were oral CLMD alone versus antidepressants, and 8 RCTs were oral CLMD with antidepressants versus antidepressants. Meta-analysis results showed that oral administration of CLMD could improve Hamilton's Depression Scale (HAMD) and the Modified Edinburgh-Scandinavian Stroke Scale (MESSS) scores, improve the Barthel index, and have a low rate of adverse reactions, but there was no significant difference in the total effective rate (*p*=0.21 > 0.05) and the National Institute of Health Stroke Scale (NIHSS) score (*p*=0.47 > 0.05) between the antidepressants group and the oral administration of the CLMD group. Oral CLMD combined with antidepressants could improve the total effective rate, HAMD, and MESSS score, but there was no significant difference in Barthel index (*p*=0.06 > 0.05) and the adverse reaction rate (*p*=0.14 > 0.05) between the two groups.

**Conclusion:**

Current evidence suggests that oral CLMD alone or with antidepressants is more effective and safer in the treatment of PSD than oral antidepressants. Due to the limitation of the quality and quantity of the included studies, more high-quality studies are needed to confirm the above conclusion.

## 1. Introduction

Poststroke depression (PSD) refers to a series of psychological and physical syndromes featured with depression, slow response, loss of interest, and other symptoms after stroke [1]. The incidence of PSD ranges from 29% to 31% [2], and it usually occurs within 1 year after stroke [3]. PSD is closely related to the poor prognosis of stroke, which leads to prolonged hospitalization, neurological recovery disorder, more loss of independent living ability, and even increased mortality [1, 4, 5]. Studies have shown that the mortality of patients with PSD is significantly higher than that of patients with stroke alone, which is 1.28–1.75 times higher, and the severity of depression is highly correlated with the mortality [6]. Antidepressants are the first choice for the treatment of PSD, including the selective serotonin reuptake inhibitor (SSRI), serotonin norepinephrine reuptake inhibitor (SNRI), noradrenergic and specific serotonergic antidepressant (NaSSA), and tricyclic antidepressants (TCAs) [1]. Although clinical studies have confirmed that antidepressants are effective for PSD [7, 8] and recommended by guidelines [9], these drugs require long-term use and are prone to dependence and many adverse reactions [10]. These negative factors may force PSD patients or clinicians to explore other treatment options. Therefore, it is crucial to provide better treatment strategies for PSD patients.

Traditional Chinese medicine (TCM) has many advantages, such as multitarget, multipathway, and strong safety, which plays an important role in the complementary and alternative therapies, and has accumulated rich experience in the practice of treating PSD [11]. According to TCM theory, the pathogenesis of PSD is mainly liver qi stagnation, accompanied by the damage to brain collaterals and imbalance of qi, blood, and Yin and Yang after stroke, and the pathological characteristic is intermingled deficiency and excess [12]. Chaihu Jia Longgu Muli decoction (CLMD), a representative prescription for the treatment of mental diseases, is composed of Chaihu (Radix Bupleuri), Longgu (Os Draconis), Muli (Concha Ostreae), Huang Qin (Radix Scutellariae), Shengjiang (Rhizoma Zingiberis Recens), Da Zao (Fructus Jujubae), Qian Dan (Miniumite), Ren Shen (Radix Ginseng), Gui Zhi (Ramulus Cinnamomi), Fuling (*Poria*), Ban Xia (Rhizoma Pinelliae), and Da Huang (Radix et Rhizoma Rhei), which has the effect of soothing liver qi stagnation, regulating qi and blood, calming the mind, and relieving fright. It is widely used in dementia, insomnia, anxiety, depression, and other mental diseases, and its effect is reliable [13]. Meanwhile, there are more and more clinical studies on the application of CLMD in PSD. Liu et al. [14] showed that CLMD can significantly improve the depression and quality of life of the patients through a randomized controlled trial, which has similar efficacy with fluoxetine, and has less side effects. Zhao et al. [15] observed that CLMD combined with antidepressant has a synergistic effect in ameliorating depression, improving the ability of daily living, and reducing inflammatory cytokines, without increasing adverse reactions. There are more and more similar reports, but it is difficult to draw a reliable conclusion due to the differences in the clinical efficacy of CLMD, research design, and course of treatment. Therefore, the purpose of this systematic review is to evaluate the efficacy and safety of CLMD in the treatment of PSD and to provide a reliable treatment option and evidence-based basis for clinical work and scientific research.

## 2. Methods

The protocol and registration information are available at https://www.crd.york.ac.uk/prospero/display_record.php?ID=CRD42021255407 (registration number: CRD42021255407). We performed this meta-analysis according to the Preferred Reporting Items for Systematic Reviews and Meta-Analyses (PRISMA) statement (Table S1).

### 2.1. Search Strategy

PubMed, the Cochrane Library, Embase, Web of Science, China National Knowledge Infrastructure, Wanfang Database, VIP Database, and China Biomedical Literature Service System (CBM) were searched by computer from the establishment of the databases to May 2021. The retrieval method adopted the combination of medical subject headings (MeSH) terms and free terms, and the English retrieval words mainly included stroke, cerebrovascular accident, depression, Chaihu Jia Longgu Muli decoction , and Chaihu Jia Longgu Muli granules (Table S2). All literature were reviewed by two investigators (Renhong Wan and Yihua Fan) independently. Any disagreement was resolved by the consultation with a third researcher (Ruiwen Song).

### 2.2. Inclusion and Exclusion Criteria

#### 2.2.1. Inclusion Criteria

Inclusion criteria were as follows:

1. Study type: randomized controlled trials (RCTs) of CLMD in the treatment of PSD
2. Diagnostic criteria: the diagnostic criteria for stroke refer to the Diagnostic Points of Various Cerebrovascular Diseases [16], for depression refer to the ones for PSD in the Classification and Diagnostic Criteria of Mental Disorders in China [17], and for TCM refer to the Diagnostic Efficacy Criteria for Diseases of TCM
3. Interventions: the treatment group was given CLMD or combined with the antidepressant, while the control group received the same antidepressant as the treatment group. (Oral preparations of Chaihu Jia Longgu Muli (CLM) included different forms such as CLMD and CLM granules. Modified CLMD referred to the addition or subtraction of no more than 3 herbs in PSD patients with different symptoms [18]).
4. Outcome indicators: the main outcome indicators are (i) total effective rate: efficacy was assessed by the reduction rate of Hamilton's Depression Scale (HAMD) score. The criteria for the efficacy of depression were recovery, score reduction rate >75%; significant effect, the reduction rate >50%; effectiveness, score reduction rate ≥25%; ineffectiveness, subtraction rate <25%; and total effective rate = (recovery number + significant effect number + effectiveness number)/total number*∗*100%; and (ii) HAMD score: secondary outcome indicators: (i) Barthel index for activities of daily living; (ii) National Institute of Health Stroke Scale (NIHSS); (iii) the Modified Edinburgh-Scandinavian Stroke Scale (MESSS); and (iv) adverse reactions rate.

#### 2.2.2. Exclusion Criteria

Exclusion criteria were as follows:

1. For repetitive studies, only the studies with the highest quality and best data were included
2. Studies with incomplete data or significant errors that cannot be resolved after contact with the author
3. Studies in which random methods or allocation concealment were evaluated as high risk
4. Studies without outcome indicators

### 2.3. Study Selection and Data Extraction

Two independent reviewers screened the literature strictly according to the inclusion and exclusion criteria, extracted the data, and cross-checked the data after completion. For literature and data with objections, two reviewers would discuss, and if there was no agreement, the third reviewer would be invited to evaluate. The extracted data included (i). basic information of the included studies: title, first author, year of publication, number of cases in each group, and baseline characteristics of patients; (ii) intervention measures and treatment course of treatment group and control group; (iii) outcome indicators; and (iv) each risk bias assessment elements in RCTs.

### 2.4. Risk of Bias Assessment

The risk of bias in the included studies was independently evaluated by two investigators, and the results were cross-checked. If there was any disagreement, it would be discussed and resolved with the third researcher. Bias risk assessment adopted the RCT bias risk assessment tool recommended in the Cochrane manual 5.1.0 [19].

### 2.5. Statistical Analysis and Data Synthesis for Meta-Analysis

RevMan5.3 software recommended by the Cochrane Collaboration was used for meta-analysis. For continuous variables, if the measurement tools and units were the same, the weighted mean difference (WMD) was used for analysis. If the measurement tools or units were inconsistent, the standard mean difference (SMD) was used. The dichotomous variables were analyzed by relative risk (RR), and 95% confidence interval (95% CI) was used for each effect size. The heterogeneity among the included results was analyzed by the *χ*^2^ test, and the magnitude of the heterogeneity was determined quantitatively by combining with *I*^2^. If *p* > 0.10 and *I*^2^ < 50%, there was no significant heterogeneity between studies, and a fixed-effect model was used for meta-analysis. If *p* > 0.10 and *I*^2^ ≥ 50%, the heterogeneity between studies was considered significant, and then, subgroup analysis or sensitivity analysis was used to explore the source of heterogeneity. After the exclusion of obvious clinical heterogeneity and methodological heterogeneity, a random-effect model was used for meta-analysis. Sensitivity analysis was used to observe the influence of single study on the combined effect size and to analyze the stability of the meta-analysis results. For main outcome indicators, if the included studies were ≥10, the funnel plot was used to qualitatively detect publication bias. Egger's and Begg's tests were used to quantitatively assess the potential publication bias.

## 3. Results

### 3.1. Characteristics of the Studies

**Figure 1:**
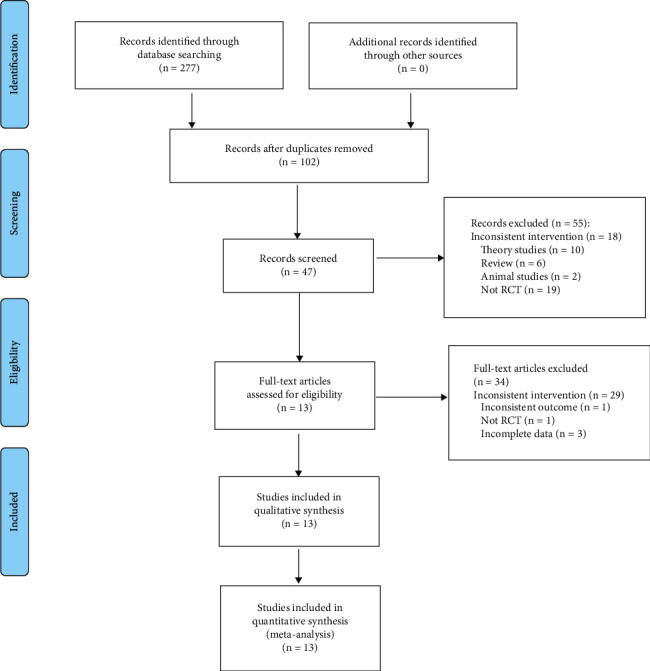
Flow diagram.

Of 277 related articles obtained by the initial search, 102 were obtained after removing the duplicates. After reading the titles and abstracts, 55 articles were excluded, and 34 were excluded after reviewing the full text in the remaining 47, so 13 studies were eligible for inclusion. The literature screening process is shown in Figure 1.

The basic characteristics of the included studies are given in Table 1. The treatment group was treated with CLMD alone or combined with antidepressants, while the control group was treated with the antidepressants, and the characteristics of the intervention measures are given in Table 2.

### 3.2. Risk of Bias Assessment

**Figure 2:**
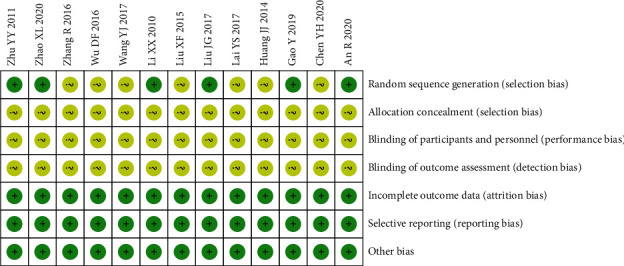
Summary of risk of bias.

The RCT risk of bias assessment tool recommended in the Cochrane Manual 5.1.0 was used to evaluate the quality of the 13 included studies, and the random sequence generation method was correctly used in 6 studies [15, 20, 22, 25, 26, 29]. None of the studies [14, 15, 20–30] mentioned the use of the blind method. All studies [14, 15, 20–30] were assessed as low risk of bias in terms of allocation concealment, incomplete outcome indicators, selective reporting, and other biases. The results are shown in Figure 2.

### 3.3. Meta-Analysis Results

Among the 13 studies included, 5 studies [14, 23, 27, 28, 30] compared oral CLMD alone with antidepressants and 8 studies [15, 20–22, 24–26, 29] compared oral CLMD combined with antidepressants with antidepressants.

#### 3.3.1. Oral CLMD Alone vs. Antidepressant

**Figure 3:**
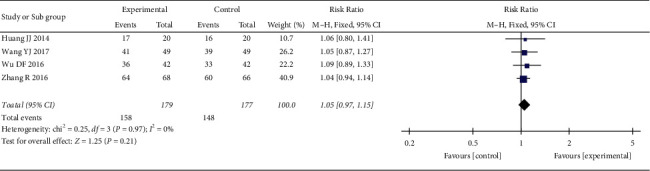
Meta-analysis of oral CLMD alone vs. antidepressant in total effective rate.

**Figure 4:**
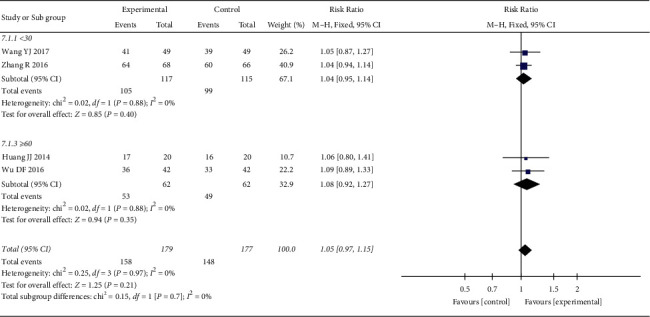
Subgroup analysis of oral CLMD alone vs. antidepressant in total effective rate.

*(1) Total Effective Rate*. 4 RCTs reported the total effective rate. The heterogeneity test showed no statistical heterogeneity (*p*=0.97; *I*^2^ = 0%). Meta-analysis of the data using a fixed-effect model showed no statistically significant difference between the treatment group and the control group ((RR = 1.05, 95% CI: 0.97, 1.15, *p*=0.21 >0.05), Figure 3). Due to the large span of treatment courses in each study, we performed a subgroup analysis based on the course. According to the course of treatment, they were divided into two subgroups: <30 days and ≥60 days. The heterogeneity test of total effective rate showed no statistical heterogeneity in <30 days (*p*=0.88; *I*^2^ = 0%) and ≥60 days (*p*=0.88; *I*^2^ = 0%) (Figure 4). Meta-analysis of data using a fixed-effect model indicated that there was no statistical difference in the two groups (<30 days (RR = 1.04, 95% CI: 0.95, 1.14, *p*=0.40 >0.05) and ≥60 days (RR = 1.08, 95% CI: 0.92, 1.27, *p*=0.35 >0.05)).

**Figure 5:**
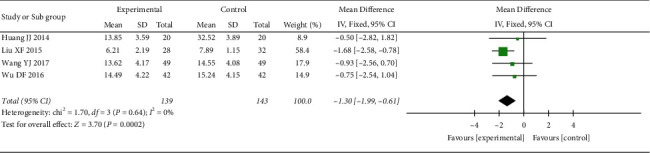
Meta-analysis of oral CLMD alone vs. antidepressant in the HAMD score.

**Figure 6:**
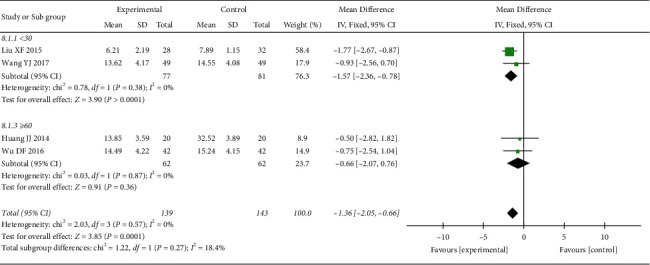
Subgroup analysis of oral CLMD alone vs. antidepressant in the HAMD score.

*(2) HAMD Score*. 4 RCTs reported the HAMD score. The heterogeneity test showed no statistical heterogeneity (*p*=0.64; *I*^2^ = 0%). Meta-analysis of the data using a fixed-effect model showed that the score of the treatment group was lower than that of the control group, and the difference was statistically significant ((MD = −1.30, 95% CI: −1.99, −0.61, *p*=0.002 <0.05), Figure 5). At the same time, we performed subgroup analysis according to the course of treatment. The heterogeneity test of the HAMD score showed no statistical heterogeneity in <30 days (*p*=0.38; *I*^2^ = 0%) and ≥60 days (*p*=0.87; *I*^2^ = 0%) (Figure 6). Meta-analysis of data was performed using a fixed-effect model, and the results indicated that the HAMD score of the treatment group was better than that of the control group after treatment in the <30 days subgroup (MD = −1.57, 95% CI: −2.36, −0.78, *p* < 0.0007), but there was no statistically significant difference between the two groups in the ≥60 days subgroup (MD = −0.66, 95% CI: −2.07, 0.76, *p*=0.36 >0.05).

*(3) MESSS Score*. 1 RCT reported the MESSS score, which could not be used for meta-analysis. Descriptive analysis showed that the treatment group was superior to the control group, and the difference between the two groups was statistically significant ((MD = −5.72, 95% CI: −8.05, −3.39), *p* < 0.00001).

*(4) NIHSS Score*. 1 RCT reported the NIHSS score, and descriptive analysis showed no statistically significant difference between the two groups ((MD = −0.37, 95% CI: −1.37, −0.63), *p*=0.47 >0.05).

**Figure 7:**
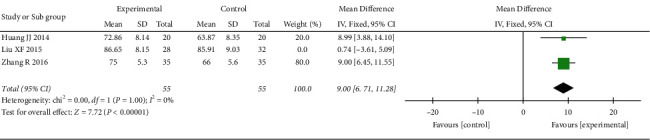
Meta-analysis of oral CLMD alone vs. antidepressant in Barthel index.

*(5) Barthel Index*. 3 RCTs reported Barthel index. The heterogeneity test suggested significant heterogeneity (*p*=0.004; *I*^2^ = 82%), so the elimination method was adopted one by one. When Liu's study [14] was excluded, heterogeneity disappeared (*p*=1.00; *I*^2^ = 0%, Figure 7), suggesting that this study was the source of heterogeneity, and a fixed-effect model was adopted after the exclusion of heterogeneity. Results showed that the score of the treatment group was better than that of the control group, and the difference was statistically significant (MD = 9.00, 95% CI: 6.45, 11.55, *p* < 0.00001).

**Figure 8:**
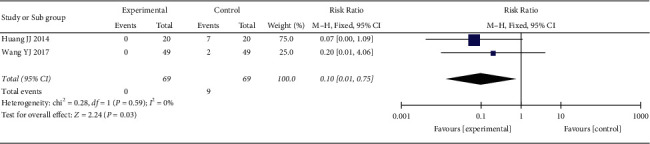
Meta-analysis of oral CLMD alone vs. antidepressant in adverse reactions rate.

*(6) Adverse Reactions Rate*. 2 RCTs reported adverse reactions rate. The heterogeneity test showed no statistical heterogeneity (*p*=0.59; *I*^2^ = 0%, Figure 8). Meta-analysis of the data using a fixed-effect model showed that the adverse reactions rate in the treatment group was lower than that in the control group, and the difference was statistically significant (RR = 0.10,95% CI: 0.01, 0.75, *p*=0.03 <0.05).

#### 3.3.2. Oral CLMD + Antidepressant vs. Antidepressant

**Figure 9:**
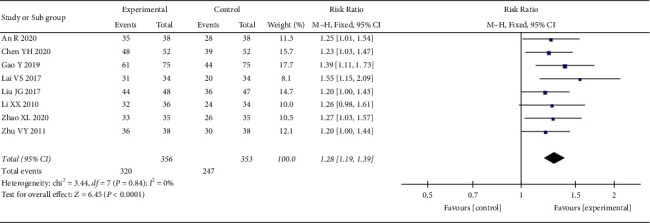
Meta-analysis of oral CLMD + antidepressant vs. antidepressant in total effective rate.

**Figure 10:**
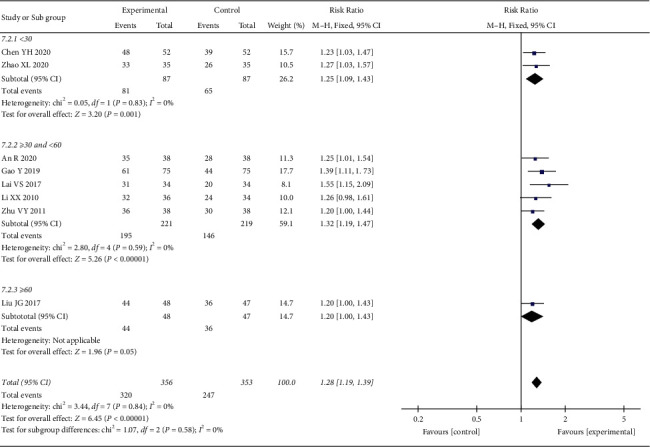
Subgroup analysis of oral CLMD + antidepressant vs. antidepressant in total effective rate.

*(1) Total Effective Rate*. 8 RCTs reported the total effective rate. The heterogeneity test showed no statistical heterogeneity (*p* = 0.84; *I*^2^ = 0%). Meta-analysis of the data using a fixed-effect model showed that the total effective rate of the treatment group was better than that of the control group, and the difference between the two groups was statistically significant ((RR = 1.28, 95% CI: 1.19, 1.39, *p* < 0.00001), Figure 9). We also conducted subgroup analysis according to the course of treatment, and the studies were divided into three subgroups: <30 days, ≥30 days and <60 days, and ≥60 days. The heterogeneity test showed no statistical heterogeneity in the <30 days subgroup (*p*=0.83; *I*^2^ = 0%) and the ≥30 days and <60 days subgroup (*p*=0.59; *I*^2^ = 0%) (Figure 10). Meta-analysis was performed on the data using a fixed-effect model, and the results showed that oral CLMD combined with antidepressants was better than oral antidepressants alone within 60 days of treatment (<30 days, (RR = 1.25, 95% CI: 1.09, 1.43, *p*=0.001) and ≥30 and <60 days (RR = 1.32, 95% CI: 1.19, 1.47, *p* < 0.00001)). There was no significant difference between the two groups after >60 days of treatment (*p*=0.05).

**Figure 11:**
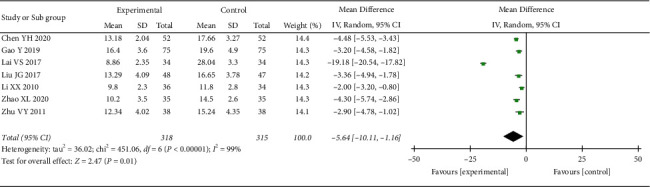
Meta-analysis of oral CLMD + antidepressant vs. antidepressant in the HAMD score.

**Figure 12:**
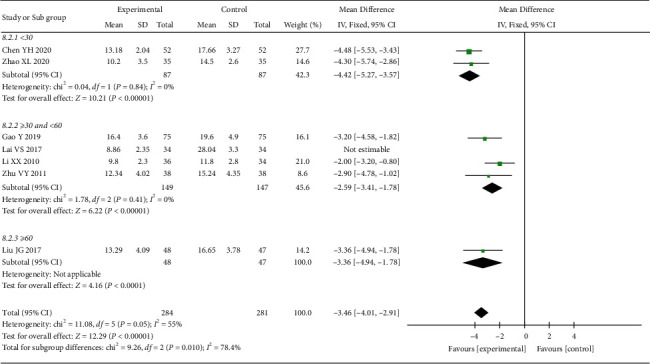
Subgroup analysis of oral CLMD + antidepressant vs. antidepressant in the HAMD score.

*(2) HAMD Score*. 7 RCTs reported the HAMD score. The heterogeneity test indicated significant heterogeneity (*p* < 0.00001; *I*^2^ = 99%). However, the confidence intervals in the forest plot were all on the left side of the invalid line, indicating that the heterogeneity among studies did not affect the results. Therefore, a random-effect model was adopted for the combination. The results showed that the HAMD score of the treatment group was better than that of the control group, and the difference was statistically significant ((MD = −5.64, 95% CI: −10.11, −1.16, *p*=0.01 <0.05), Figure 11). To explore the source of heterogeneity, we performed subgroup analysis according to the course of treatment. The heterogeneity test result of the subgroup (≥30 and <60 days) was *p* < 0.00001 and *I*^2^ = 99%, indicating significant heterogeneity. When Lai's study was excluded [24], the heterogeneity disappeared (*p*=0.41; *I*^2^ = 0%), suggesting that this study was the source of heterogeneity. A fixed-effect model was used for meta-analysis of the data, and the results showed that HAMD scores in the treatment groups were all better than those in the control groups, with statistically significant differences in the <30 days subgroup (MD = −4.42, 95% CI: −5.27, −3.57, *p* < 0.00001), the ≥30 and <60 days subgroup (MD = −2.59, 95% CI: −3.41, −1.78, *p* < 0.00001), and the ≥60 days subgroup (MD = −3.36, 95% CI: −4.94, −1.78, *p* < 0.00001) (Figure 12).

*(3) MESSS Score*. 1 RCT reported the MESSS score, which could not be used for meta-analysis. Descriptive analysis showed that the treatment group was superior to the control group, and the difference between the two groups was statistically significant (MD = −5.26, 95% CI: −7.55, −2.97, *p* < 0.00001).

**Figure 13:**
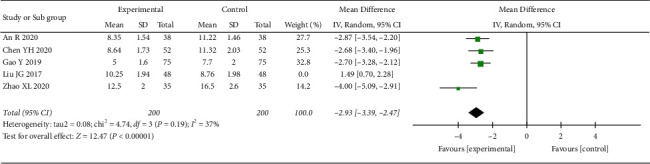
Meta-analysis results of oral CLMD + antidepressant vs. antidepressant in the NIHSS score.

*(4) NIHSS Score*. 4 RCTs reported the NIHSS score. The heterogeneity test indicated significant heterogeneity (*p* < 0.00001; *I*^2^ = 99%). A one-by-one elimination method was used to analyze the source of heterogeneity. When Liu and Zhang's study [26] was excluded, the heterogeneity was significantly reduced (*p*=0.19; *I*^2^ = 37%, Figure 13), suggesting that the study was the source of heterogeneity, and a fixed-effect model was adopted after the exclusion of heterogeneity. Results showed that the NIHSS score of the treatment group was better than that of the control group, and the difference was statistically significant (MD = −2.93, 95% CI: −3.39, −2.47, *p* < 0.00001).

**Figure 14:**
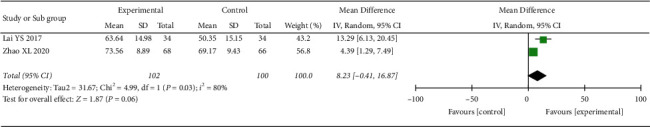
Meta-analysis results of oral CLMD + antidepressant vs. antidepressant in Barthel index.

*(5) Barthel Index*. 2 RCTs reported Barthel index, and the heterogeneity test suggested significant heterogeneity (*p*=0.03; *I*^2^ = 80%). Due to the small number of the included studies that could not be further analyzed and the study results were all on the side of the invalid line, a random-effect model was adopted. The result showed that there was no statistically significant difference between the two groups ((MD = 8.23, 95% CI: −0.41, −16.87, *p*=0.06 >0.05), Figure 14).

**Figure 15:**
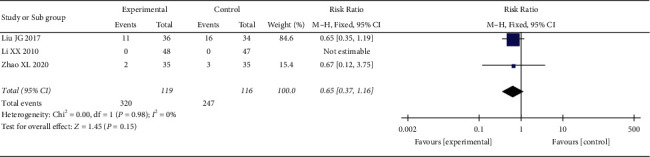
Meta-analysis results of oral CLMD + antidepressant vs. antidepressant in adverse reactions rate.

*(6) Adverse Reactions Rate*. 3 RCTs reported the rate of adverse reactions. The heterogeneity test showed no statistical heterogeneity (*p*=0.98; *I*^*2*^ = 0%, Figure 15). Meta-analysis of the data using a fixed-effect model showed that there was no significant difference between the two groups (RR = 0.65, 95% CI: 0.37, 1.16, *p*=0.15 >0.05).

### 3.4. Sensitivity Analysis

Sensitivity analysis of the above indicators was conducted by the one-by-one elimination method, and changes of the effect size and *p* value were observed after the one-by-one exclusion of the included studies. The results showed that the effect size of outcome indicators did not change significantly, suggesting that the results of the meta-analysis were reliable and stable.

### 3.5. Publication Bias

Egger's test and Begg's test were used to evaluate whether there was publication bias in the main outcome indicators. For oral CLMD alone vs. antidepressant, no evidence of publication bias was found in the effective rate (Egger's test *p*=0.7165 >0.05, Begg's test *p*=0.382 >0.05) as well as the HAMD score (Egger's test *p*=0.6926 >0.05, Begg's test *p*=1.6918 >0.05). As for oral CLMD + antidepressant vs. antidepressant, there was publication bias in the effective rate (Egger's test *p*=0.002 <0.05, Begg's test *p*=0.0354 <0.05) and the HAMD score (Egger's test *p* < 0.0001, Begg's test *p*=1.9285 >0.05).

## 4. Discussion

In this study, a meta-analysis of 13 RCTs of CLMD in the treatment of PSD showed that the following. (1) In terms of total effective rate, we found that CLMD combined with antidepressants was more effective than antidepressants alone, while there was no difference between CLMD and antidepressants alone; (2) HAMD is the most commonly used in the assessment of depressive symptoms, and both CLMD alone and CLMD with antidepressants were better than antidepressants alone in reducing HAMD scores. Depending on the course of treatment, we found different conclusions. When the course of treatment was <30 days, oral CLMD was more effective than antidepressants alone. When the course of treatment was ≥60 days, the efficacy of oral CLMD was comparable to that of antidepressants alone. Whether the treatment course was short or long, the efficacy of CLMD combined with antidepressants was better than that of the antidepressants group, indicating that the treatment of CLMD combined with antidepressants was more conducive to improving the clinical efficacy; (3) MESSS and NIHSS are the international major indicators for the evaluation of neurological function recovery after stroke, which are of great significance for the judgment of postoperative recovery after stroke. Clinical observation shows that MESSS and NIHSS have good predictive validity for the prognosis of stroke and are significantly correlated with Barthel index [31]. The higher the score is, the lower the BI value is [32]. Due to the limited number of the included studies, only the NIHSS scores of oral CLMD combined with antidepressants were meta-analyzed, and the results showed that the combined treatment group was superior to the antidepressant group; (4) Barthel index is an indicator to test the independent living ability of patients, which can reflect the degree of nursing need of the patients. Barthel index also can be used to evaluate the functional recovery of PSD patients. As the score of specific items of Barthel index was not reported in the included studies, it was impossible to objectively evaluate the specific impact of CLMD alone or CLMD + antidepressant on the independent living activities of PSD patients. The results showed that the BI score of oral CLMD alone was better than that of the antidepressant group, indicating that CLMD was positive and effective in improving the independent living ability of PSD patients, but there was no significant difference between oral CLMD combined with antidepressants and the antidepressant group, which may be related to the course of treatment; (5) in terms of adverse reactions, there were a total of 5 RCTs in our study that described adverse reactions, among which two RCTs were about CLMD alone vs. antidepressant, which reported that no adverse reactions occurred in the treatment group, while the adverse reactions in the control group included insomnia, gastrointestinal discomfort, dizziness, and headache. The remaining three RCTs were related to CLMD + antidepressant vs. antidepressant, among which one RCT reported no adverse reactions in the treatment group and the control group, while two RCTs reported no significant differences in adverse reactions between the treatment group and the control group, including abnormal blood and urine routine, abnormal liver function, insomnia, and digestive tract discomfort. It is seen that CLMD does not increase the risk of adverse reactions, but it does not reduce the side effects of depression; and (6) we conducted sensitivity analysis on all outcome indicators through the one-by-one exclusion method, and the results showed that our meta-analysis was robust.

Stroke is an important social psychological factor leading to depression. Neurological dysfunction and long-term disability caused by stroke lead to the psychological stress response, which brings about psychological imbalance [1]. Depression hinders the recovery of the neurological function after stroke. Antidepressant treatment can not only relieve the symptoms of depression but also promote the physical recovery of stroke patients, which is far more vital than the treatment of depression itself [33].

After thousands of years of exploration, Chinese medicine has advantages in the treatment of mental disorders [34–36]. CLMD is one of the most common prescriptions used in the treatment of mental diseases in TCM. Clinical studies have found that CLMD has the effect of psychotropic drugs and have shown a significant antidepressant effect on animal models [37, 38]. It can regulate the hypothalamopituitary-adrenal system dysfunction by preventing the dopaminergic and serotonergic transmission in the prefrontal cortex [39] and upregulate the expression of the brain-derived neurotrophic factor (BDNF) to alleviate the depression-like state induced by chronic stress [37]. It also has immediate and long-lasting antidepressant effects by enhancing BDNF expression in the hippocampus [38]. Chaihu (Radix Bupleuri) and Huang Qin (Radix Scutellariae) are the key drugs in many prescriptions for mental disorders. Modern pharmacological studies have found that they can reduce neuroinflammation [40] and neuronal apoptosis [41] and increase the concentration of the nerve growth factor and BDNF [42]. Baicalin in Huang Qin (Radix Scutellariae) can inhibit inflammation [43] and promote nerve regeneration [44, 45]. Baicalin also has an antidepressant-like effect [46], which is associated with the increase of BDNF in the hippocampal region [44].

Limitations of this study are as follows: some of the included studies seldom describe the specific operation of the allocation concealment and blind method, and there may be selectivity bias and measurement bias; all the studies are from China, and there may be regional restrictions; due to the particularity of TCM decoction, the composition and dosage of CLMD in the study were different, which may have a certain influence on the results of the study.

## 5. Conclusion

Current evidence supports the efficacy of CLMD in PSD patients, which can not only improve depressive symptoms but also promote the recovery of neurological and limbs functions in stroke patients. The efficacy of CLMD alone is no less than that of antidepressants, and there are fewer adverse reactions. In addition, CLMD alone was more effective when treatment was less than 30 days, while oral CLMD combined with antidepressants was more effective than antidepressants alone in both short- and long-term treatment.

## Figures and Tables

**Table 1:** Basic characteristics of the included studies.

Study cohort	No. (T/C)	Gender		Age		Course (day)	Outcome
		T	C	T	C		
An [20]	38/38	22/16	20/18	57.03 ± 7.33	56.23 ± 7.26	30	①④
Chen et al. [21]	52/52	30/22	29/23	49.6 ± 3.3	49.1 ± 3.6	21	①②④
Gao and Zhang [22]	75/75	30/45	33/42	66.5 ± 11.3	69.5 ± 12.0	30	①②④
Huang [23]	20/20	13/7	13/7	64.80 ± 7.08	65.30 ± 6.89	60	①②⑤⑥⑦
Lai et al. [24]	34/34	18/16	23/11	58.2 ± 5.8	62.1 ± 6.9	56	①②⑤
Li [25]	36/34	17/19	15/19	—	—	56	①②⑦
Liu and Yang [26]	48/47	26/22	26/21	48.35 ± 6.24	48.35 ± 6.24	63	①②④⑦
Liu et al. [14]	28/32	13/15	15/17	65.4 ± 8.7	63.7 ± 9.3	28	②④⑤
Wang and Li [27]	49/49	36/13	35/14	59.63 ± 5.27	60.05 ± 5.69	28	①②③⑦
Wu [28]	42/42	23/19	21/21	59.78 ± 7.82	60.54 ± 8.04	90	①②⑥
Zhao et al. [15]	35/35	20/15	21/14	60.4 ± 4.5	60.5 ± 4.3	28	①②④⑤⑦
Zhu [29]	38/38	24/14	26/12	46.34 ± 10.97	45.28 ± 11.26	42	①②③
Zhang et al. [30]	68/66	29/39	19/47	65.91 ± 10.442	68.12 ± 9.731	14	①②④⑤

Note: ①, efficiency; ②, HAMD; ③, MESSS; ④, NIHSSS; ⑤, Barthel; ⑥, CSS; ⑦, adverse reactions rate; —, unclear.

**Table 2:** Characteristics of the interventions.

Study	Interventions of the treatment group		Interventions of the control group	Days
	CLMD	Antidepressants	Antidepressants	
An [20]	Chaihu Jia Longgu Muli decoction, 100 ml bid	Fluoxetine hydrochloride capsules 20 mg qd	Fluoxetine hydrochloride capsules 20 mg qd	30
Chen et al. [21]	Chaihu Jia Longgu Muli decoction, 100 ml bid	Paroxetine 10 mg qd, 10 mg was added after 1 week	Paroxetine 10 mg qd, 10 mg was added after 1 week	21
Gao and Zhang [22]	Chaihu Jia Longgu Muli decoction, 100 ml bid	Flupentixol 0.5 mg bid and melitracen 10 mg bid	Flupentixol 0.5 mg bid and melitracen 10 mg bid	30
Huang [23]	Chaihu Jia Longgu Muli decoction, 100 ml bid	None	Fluoxetine hydrochloride 20 mg qd	60
Lai et al. [24]	Chaihu Jia Longgu Muli decoction, 125 ml bid	Flupentixol and melitracen tablets (flupentixol 0.5 mg and melitracen 10 mg) 2#qd	Flupentixol and melitracen tablets (flupentixol 0.5 mg and melitracen 10 mg) 2#qd	56
Li [25]	Chaihu Jia Longgu Muli decoction, 100 ml bid	Fluoxetine hydrochloride 20 mg qd	Fluoxetine hydrochloride 20 mg qd	56
Liu and Yang [26]	Chaihu Jia Longgu Muli decoction, 100 ml bid	Paroxetine 10 mg qd, 10 mg was added after 1 week	Paroxetine 10 mg qd, 10 mg was added after 1 week	63
Liu et al. [14]	Chaihu Jia Longgu Muli decoction, 100 ml bid	None	Fluoxetine hydrochloride tablets 20 mg qd	28
Wang and Li [27]	Chaihu Jia Longgu Muli decoction, 100 ml bid	None	Fluoxetine hydrochloride tablets 20 mg qd	28
Wu [28]	Chaihu Jia Longgu Muli decoction, bid	None	Fluoxetine hydrochloride 20 mg qd	90
Zhao et al. [15]	Chaihu Jia Longgu Muli decoction, bid	Fluoxetine hydrochloride capsules 20 mg bid	Fluoxetine hydrochloride capsules 20 mg bid	28
Zhu [29]	Chaihu Jia Longgu Muli decoction	Conventional Western medicine	Conventional Western medicine	42
Zhang et al. [30]	Chaihu and Longgu Muli granules, 100 ml bid	None	Citalopram 20 mg qd	14

## Data Availability

The data used to support the findings of this study are available from the corresponding author upon request.

## Abbreviations

PSD: Poststroke depression
SSRI: Selective serotonin reuptake inhibitor
SNRI: Serotonin norepinephrine reuptake inhibitor
NaSSA: Noradrenergic and specific serotonergic antidepressant
TCAs: Tricyclic antidepressants
TCM: Traditional Chinese medicine
CLMD: Chaihu Jia Longgu Muli decoction
CBM: China biomedical literature service system
RCTs: Randomized controlled trials
CLM: Chaihu Jia Longgu Muli
HAMD: Hamilton's Depression Scale
NIHSS: National Institute of Health Stroke Scale
MESSS: Modified Edinburgh-Scandinavian Stroke Scale
WMD: Weighted mean difference
SMD: Standard mean difference
RR: Relative risk
BDNF: Brain-derived neurotrophic factor.

## Supplementary Materials

Supplementary Materials Table S1. The PRISMA checklist. Table S2. Search strategy in PubMed database.
