# Supplementary material for: Efficacy and Safety of Chaihu Jia Longgu Muli Decoction in the Treatment of Poststroke Depression: A Systematic Review and Meta-Analysis
Source: Evid Based Complement Alternat Med. 2021 Aug 19;2021:7604537. doi: 10.1155/2021/7604537 (PMC8397549; doi:10.1155/2021/7604537)
Supplement: Supplementary Materials — Table S1. The PRISMA checklist. Table S2. Search strategy in PubMed database. [file 7604537.f1.zip › 7604537.f1/Table S2 Search strategy in PubMed database..docx]

| Number | Search terms |
| --- | --- |
| #1 | Stroke [MeSH] |
| #2 | Cerebrovascular Accident [Title/Abstract] |
| #3 | Cerebrovascular Apoplexy [Title/Abstract] |
| #4 | Brain Vascular Accident [Title/Abstract] |
| #5 | Cerebrovascular Stroke [Title/Abstract] |
| #6 | Apoplexy [Title/Abstract] |
| #7 | Acute Cerebrovascular Accident [Title/Abstract] |
| #8 | #1 OR #2 OR #3 OR#4 OR#5 OR #6 OR #7 |
| #9 | Depression [MeSH] |
| #10 | Depressive Symptom [Title/Abstract] |
| #11 | Emotional Depression [Title/Abstract] |
| #12 | #9 OR #10 OR #11 |
| #13 | Chaihu-jia-Longgu-Muli Decoction [Title/Abstract] |
| #14 | Chaihu-jia-Longgu-Muli granules [Title/Abstract] |
| #15 | Chaihu jia Longgu Muli [Title/Abstract] |
| #16 | CLM [Title/Abstract] |
| #17 | #13 OR #14 OR #15 OR #16 |
| #18 | #8 AND #12 AND #17 |
